# Policy options to improve leadership of middle managers in the Australian residential aged care setting: a narrative synthesis

**DOI:** 10.1186/1472-6963-10-190

**Published:** 2010-07-06

**Authors:** Yun-Hee Jeon, Nicholas J Glasgow, Teri Merlyn, Emily Sansoni

**Affiliations:** 1 The Australian Primary Health Care Research Institute, The College of Medicine, Biology and Environment, The Australian National University, Building 62, Mills Rd, Canberra, Australia; 2 Sydney Nursing School, The University of Sydney, Mallett St., Camperdown, Australia; 3 Medical School, The College of Medicine, Biology and Environment, The Australian National University, Frank Fenner Building 42, Canberra, Australia

## Abstract

**Background:**

The prevalence of both chronic diseases and multi-morbidity increases with longer life spans. As Australia's population ages, the aged care sector is under increasing pressure to ensure that quality aged care is available. Key to responding to this pressure is leadership and management capability within the aged care workforce. A systematic literature review was conducted to inform the policy development necessary for the enhancement of clinical and managerial leadership skills of middle managers within residential aged care.

**Methods:**

Using scientific journal databases, hand searching of specialist journals, Google, snowballing and suggestions from experts, 4,484 papers were found. After a seven-tiered culling process, we conducted a detailed review (narrative synthesis) of 153 papers relevant to leadership and management development in aged care, incorporating expert and key stakeholder consultations.

**Results:**

• Positive staff experiences of a manager's leadership are critical to ensure job satisfaction and workforce retention, the provision of quality care and the well-being of care recipients, and potentially a reduction of associated costs.

• The essential attributes of good leadership for aged care middle management are a hands-on accessibility and professional expertise in nurturing respect, recognition and team building, along with effective communication and flexibility. However, successful leadership and management outcomes depend on coherent and good organisational leadership (structural and psychological empowerment).

• There is inadequate preparation for middle management leadership roles in the aged care sector and a lack of clear guidelines and key performance indicators to assess leadership and management skills.

• Theory development in aged care leadership and management research is limited. A few effective generic clinical leadership programs targeting both clinical and managerial leaders exist. However, little is known regarding how appropriate and effective they are for the aged care sector.

**Conclusions:**

There is an urgent need for a national strategy that promotes a common approach to aged care leadership and management development, one that is sector-appropriate and congruent with the philosophy of person-centred care now predominant in the sector. The onus is on aged care industries as a whole and various levels of Government to make a concerted effort to establish relevant regulation, legislation and funding.

## Background

In 2005, the Australian aged care sector employed 1.3 percent of the workforce and was ninth in total employment, making it a major contributor to the economy [1]. The aged care sector shares many workforce issues common to the wider nursing sector and competes with acute and primary sectors in a shrinking pool of qualified nurses [2,3]. There are also concerns unique to this sector, in particular registered nurse staffing ratios or skill-mix [4,5]. Residential aged care (RAC) has gone from a cottage industry to a formal aged care service for clients with complex health issues, requiring sophisticated knowledge and expertise from care staff [3,6]. Most personal care in residential aged care facilities (RACFs) is provided by assistants in nursing or personal care assistants, yet they are more likely to have limited knowledge and skills in complex care needs. According to the latest national survey of the Australian RAC workforce, in 2007 64 percent of care staff in RACFs were personal carers, a five percent increase in this workforce demographic over four years [4]. This places greater emphasis on the quality of clinical supervision and leadership capabilities among middle managers, which are found to be in short supply across the profession [2,4].

Australia's ageing population, together with the increasing prevalence of chronic diseases and multi-morbidity, are placing the aged care sector under pressure, which impacts directly on the RAC workforce [7,8]. Strategies targeted at those in leadership and management positions of the RAC workforce will contribute to optimising its effectiveness. The National Aged Care Workforce Strategy 2005 provided a strategic approach focused on long-term structural reform in the Australian sector [1]. The Strategy identified actions to be taken and key participants for desired outcomes in developing sustainable workforce leadership and effective management that are yet to be enacted.

This review focuses on the central question of 'what policy and system solutions are necessary to build capacity for sustainable workforce leadership and effective management in residential aged care?' *The aim is to provide policy options, strategies and recommendations for the enhancement of workforce leadership and management within the residential aged care sector, to promote and maintain best practice.*

### Definitions and scope of the review

#### Leadership and management

Evidence suggests that the debate on leadership versus management and their relationship to each other is ongoing and further work needs to be done to elucidate their distinctive parameters and characteristics. Some argue that leadership is part of the management function, or vice versa, while others believe they have different goals and are operationalised in a range of ways. A systematic review of research in health services leadership found much confusion between the constructs of leadership and of management, with 'old management programmes being repackaged as leadership' [9]. Our position in this review is that there is a distinction between the two: i) whilst management can be prescribed, leadership is about the development and building of social capital and some of its elements need to be recognised as 'emergent' (i.e. emerging organically out of facilitative environments) rather than being prescribed [9]; and ii) leadership is an external focus with future vision, whilst management's internal focus is on immediate needs [10]. Distinctions are also made through the identification of elements of leadership and management, shown in Table 1. With this distinction in mind, those systems and protocols within which leadership manages the human and material resources under its charge are the testing grounds of an individual's leadership capability and management skills. In this review we also accept there are overlapping factors between leadership and management, as shown in Table 1, and between the prescribed and emergent capabilities required by each.

**Table 1:** Elements of leadership and management (pp.13-14) [11]

Elements of Leadership	Overlapping elements of leadership and management	Elements of management
inspiration, transformation, direction, trust, empowerment, creativity, innovation and motivation	communication, decision-making, integrity, role model, negotiation, professional competence and setting standards	delegation, performance, planning, accountability, finance, teamwork and team building, monitoring and evaluating, formal supervision and control

What is clear from the examination of literature on leadership and management is that both need to be treated as equally important and integral to the creation of an enabling and supportive environment to optimise workforce capacity. "Leadership and management should be integrated and complementary" (p.13), so that leadership is reflected in management roles at all levels [11]. This has influenced the way the current paper makes reference to leadership and management. For the most part, the term 'leadership' is used throughout the paper when interrogating the literature which refers to 'leadership and management' synonymously. While there is good evidence to suggest that leadership and management are two distinct concepts and thus ought to be considered separately, to have selected review materials from this perspective would have severely limited the utility of the review findings. Since 'leadership/leader' and 'manager/management' have been used interchangeably in the majority of literature reviewed, it was considered useful to adhere to this perspective to obtain a fuller picture of the concepts of interest.

#### Types of leaders/managers

Terminologies and their definitions describing managers vary in the literature. Borrill et al. [12] make distinctions between senior management and immediate management. A senior manager is referred to as the person responsible for setting the strategic direction for the organisation and immediate management is referred to as the person responsible for line management and staff supervision [12]. Most literature uses 'executive management' as somewhat equivalent to 'senior management' while 'middle management' is equivalent to 'immediate management'. *'Leadership' in this report consists of clinical leadership and managerial leadership as middle management*. Target populations therefore include administrative, managerial and supervisory positions, such as directors or assistant directors of nursing or care managers whose roles involve the assessment of residents' health, the development of treatment plans and the supervision of other nursing staff or care workers, as well as human resource management. This way of defining middle management for the review allowed us to exclude line managers and lower-level supervisors with no managerial responsibility for a unit or facility, and senior executive managers who have no direct and on-going interactions with staff in general. Consequently, for this review we combined the concepts of leadership with middle management, as it is about managerial leadership of middle managers who also have a clinical management role, which are most readily evident and employed interchangeably within the residential aged care sector.

## Methods

We utilised a systematic literature review and narrative synthesis--a process of synthesising primary studies to explore heterogeneity descriptively rather than statistically--***to produce evidence for developing policy solutions and decision making for policy and decision makers and service providers ***[13]. Unlike systematic reviews conducted to garner concept knowledge supported by high quality evidence (namely 'review for knowledge support'[13]), this review aimed to develop recommendations for action that are context and time sensitive (namely 'review for decision support'[13]). Therefore, a synthesis of existing research evidence was conducted, combined with expert opinions in the field of inquiry (consultations) [13]. The following four methodological steps were not undertaken in a linear fashion, rather there was a dynamic interplay between them.

### Search terms and search engines

Leadership and management related terms ('leadership', 'leaders', 'management', 'managers', 'workforce', 'organisational development', 'organisational theory', 'organisational structure', 'organisational behaviour', or 'change theory') were combined with the health service related terms using the 'AND' operator ('health', 'health services', 'health care', 'residential aged care', 'nursing homes', 'nursing education' or 'long-term care'). We searched for both black and grey literature using an electronic database search (e.g., Medline, PsycINFO, CINAHL, PubMed, and Cochrane Library), hand searching of specialist journals, Google, and snowballing (the scanning of reference lists from identified studies and suggestions from experts in the field).

### Selection and appraisal of studies for their relevance

We included materials discussing the provision of effective workforce leadership and management in health care, with a particular focus on RAC settings in theoretical and conceptual elements, organisational culture, organisational development, policy guidelines, influencing factors (barriers and facilitators), leadership development, and effective leadership models or programs. Materials were excluded if they focused largely on disease/illness management, clinical pathways, clinical management, or service delivery/care models where the main emphasis was on clinical outcomes associated with patient care management or practice. If the materials focused on both clinical management and workforce issues they were included. Materials published before 1997, not in English and unpublished theses/dissertations were excluded.

Table 2 shows three initial tiers resulting from 4,484 original findings. Subsequently, 767 potentially relevant articles were classified into three categories (Tiers 4 and 5) including:

**Table 2:** Number of references by source and tier

Sources	Tier 1	Tier 2	Tier 3
Electronic database	3044	766	499
Hand search	285	129	122
Relevant references	79	79	74
Grey literature	1076	73	72
Total	4484	1047	767

• Q1: Leadership and management issues related to health care other than aged care, e.g., acute, sub-acute, community and primary health care (n = 428)

• Q2: Leadership and management issues related to aged care, residential aged care, nursing homes or long-term care (n = 226)

• Q3: Leadership models, programs or theories/frameworks (n = 113)

For Q1, only systematic reviews were included (Tier 5). Of the remaining 305 full-text papers, 158 were selected for in-depth examinations and inclusion in summary sheets (Tier 6). 153 papers were included in the final report (Tier 7).

The first author conducted Tiers 1-3. Tiers 4-7 were initially conducted by the second and third author and the first author verified their relevance and appropriateness for inclusion/exclusion in the review. Initially, the National Institute for Health and Clinical Excellence (NICE) Guidelines[14] were employed to rate the quality of evidence, feeding into the selection of final papers. However, a dearth of research using a rigorous experimental design rendered this approach inappropriate. The review team chose an inclusive approach that reflected the diversity of the literature, which proved to be useful in examining the complex issue of the aged care workforce. Given the paucity of research in Australian aged care settings, it was necessary to include all relevant Australian studies.

### Consultations

Consultations with a reference group, international experts, and directors/care managers were conducted. Reference group members consisted of people from key Australian government, non-government and consumer organisations for aged care and dementia, aged care providers and relevant professional organisations, as well as researchers/academics. The group provided expert advice on the direction and conduct of methods and actions and the development of literature review outcomes, through emails and meetings. Face-to-face meetings were conducted with experts in the United Kingdom, including senior academics and managers involved in leadership and management development or research. They suggested new references for inclusion, and key findings and recommendations from the preliminary report were confirmed as valid in light of existing literature and relevance to international contexts. In addition, managers from RACFs were invited to a meeting and verified the key findings and recommendations and provided additional viewpoints and suggestions.

### Synthesis and interpretation

A synthesis founded on research documents was guided by the following review questions.

1. What does the literature tell us about clinical and managerial leadership within management in relation to the residential aged care workforce?

2. What are the essential characteristics and the influencing factors (e.g., individual, policy and system related) necessary to sustain effective workforce leadership within management?

3. What are the best models in developing sustainable workforce leadership within management in residential aged care?

(Please refer to the full report for a detailed description of the methods [15].)

## Results

A paucity of work has been done in Australia in terms of managerial leadership development for middle management in the aged care sector. The initial search of literature indicates most studies have been acute care setting oriented and overseas based, largely from the UK and North America. Theory development in aged care leadership research is limited, resulting in attempts to source a suitable model from health management discourse. The review found scant evidence of rigorous research demonstrating the relevance of leadership theory and models for education, training and development in the aged care sector.

The following provides a summary of the key findings pertinent to the review questions. Notably, it was necessary to go beyond the residential aged care literature, due to the patchy nature of available evidence. A detailed description of the review findings can be found in the full report [15].

### Positive staff perception of a manager's leadership

Positive staff perception of a manager's leadership and support is associated with improved job satisfaction and workforce retention [12,16], with its corollaries in higher care quality, the well-being of care recipients [17,18] and a reduction of associated costs [19,20]. A review by Gagnon et al. [16] found that key factors in nurses' intentions to leave their jobs included low professional support and poor recognition, with both related to poor leadership conduct. The outcomes of programs focused on developing nurse manager leadership skills included significant improvements in staff perceptions and intentions to remain.

As the interface between the executive leadership and the care workforce, the nurse manager role is pivotal in communicating the organisational values and protocols that generate a healthy workplace culture through staff satisfaction and organisational commitment [21]. Education and training programs focused on improving the quality of leadership and management through engaging managers in staff training programs have been shown to increase staff productivity and performance, both improving care quality [22] and therefore the core function of organisational performance [23]; although this correlation is yet to be unequivocally confirmed [24].

The quality and stability of leadership and management is often integral to the success of costly staff training interventions. The persistently high turnover of both leadership and direct care staff presents RACFs with the pernicious costs resulting from lower care quality [22,25] and the high costs of perennial turnover [26,27]. A multi-centre Canadian-Australian study estimated the cost of Australian registered nurse (RN) turnover to be between AU$16,634 and AU$19,663 [27]. Investment in good leadership has been demonstrated to be cost-effective, thereby reducing turnover [20].

### The essential individual attributes of managerial leadership

Common essential individual attributes of leadership identified in virtually every consultative study are flexibility, hands-on accessibility and effective communication, professional expertise in nurturing respect and recognition and empowering team building capabilities. All studies on essential leadership attributes emphasise various facets of a common range of highly desirable attributes of individual leadership that include openness, enthusiasm, respect and consideration, role modelling, mentoring and supervision [28,29], an actively nurturing, supportive and motivational style [30], authoritative style and emotional intelligence [24,29], and organisational agility and political astuteness [31]. These attributes all contribute to an essential function of leadership, that of peer and organisational networking [31]. A positive attitude is also considered a generally desirable leadership attribute for the nurse manager and found to produce more satisfied staff and patients and less staff turnover [32].

Leadership style has a dramatic effect on staff motivation for productivity [33], stress, job satisfaction and workforce retention [34]. Leaders who inspire and empower others are more likely to model values and ethics that are congruent with their personal manner and professional practice [24,35-37] and be aligned with the organisational values and expectations they transmit [25,38,39]. To be an effective team builder, leaders need not only possess those personal attributes identified above, but also demonstrate highly developed skills in self-management and self-awareness [40].

The complexity of RAC leadership roles requires both common and unique individual attributes, some of which may be innate and emergent when nurtured in situ by mentoring (for which leadership skills are essential), and others that need to be developed in training [9,41]. Although desired leadership attributes and core competencies are listed in a number of papers [9,31], these are generic and there is little in the way of hard evidence for these knowledge and skill sets or how best to develop the effective leadership and management necessary for the future of aged care. Key performance indicators to assess aged care leadership and management skills do not exist.

Recent research in leadership has revealed a mostly ad hoc, rather than deliberate, approach to planning, with effective leadership in many countries more often a combination of natural ability, individual professional ambition and good fortune [9,42,43]. At the same time, nursing leadership has emerged as a central factor in many of the problems besieging the RAC sector and alternatives are in varying stages of development [44]. Others are working on models to identify and develop promising individuals as leaders that will inform educational curricula to build leadership competencies [31,37]

### Organisational leadership

Successful leadership outcomes depend on organisational leadership that enables leaders to feel confident they have sufficient resources at their disposal to ensure the delivery of high quality care and sufficient support for staff [22,24,29]. Resources essential to the effective conduct of leadership include adequate skill mix of staff [45,46], clear HR practices and administrative support [12,16,47], free flow of information and communication policies [21,28,48], and attractive incentives/rewards and career pathways [35,49]. Combined, these elements produce an organisational coherence providing the structural empowerment (i.e. resources) and psychological empowerment (i.e. culture and protocols) essential to the conduct of effective leadership [50].

When an organisation provides coherent structural and psychological frameworks in which leadership and management function effectively, there are positive outcomes for both care quality and staffing [19,22,44]. Whilst not always made explicit in the variables, organisational leadership often emerges as a central factor in the outcomes of interventions, obstructing or diffusing intended effects through lack of effective leadership [51], instability [17,35,38], incoherence [52] or inadequate resources [53]. Organisational investment in leadership roles through training and supportive systems is seen to be highly cost effective in the improvement of outcomes across the spectrum and is strongly recommended [16,20,43,54].

### Theory development in aged care leadership research

Theory development in aged care leadership is limited, and almost non-existent in RAC, resulting in the adoption of models sourced from business management discourse. Of these, the transformational model appears to have the most evidential support to date [29,55]. This model proposes a dynamic, inspirational leader who inspires followers by example and motivational empowerment, and is often paired with the transactional construct of reward and reinforcement. However, there is also recognition of its alien origins and awkward fit in the much more complex healthcare environment, where collaborative, interdisciplinary teams are more egalitarian in structure [9,10,39]. Shared governance is a leadership model that is being explored for suitability in general healthcare practice settings [56,57] and in RAC through more holistic management structures [29,44]. Shared governance proposes a distributed or dispersed leadership that requires an organisational framework in which individuals are sufficiently supported and trained to work collaboratively in teams, but does not offer any articulated theory of leadership per se. Evidently, there is an urgent need for further theory development in aged care leadership research.

### Leadership preparation and support in aged care practice

Many middle managers and leaders working in aged care have limited opportunities to prepare for these roles and lack clear, congruent guidelines to support them in the roles' responsibilities [41,54]. High staff turnover, including amongst the leadership [20,25,35], absenteeism and shortages make sustained outcomes for quality improvement interventions problematic [17]. These conditions, combined with the perennial demands of government licensing and regulation with which RACFs need to comply, find RAC senior and middle managers often trapped in a cycle of crisis management with little energy or time for professional development [10]. Current trends toward Person Centred Care and interdisciplinary collaboration require a new range of leadership capabilities and management skills, which come into constant conflict with existing hierarchies and structures [48]. When a care staff culture change skills program also has organisational support from, and engages with, the leadership, the outcomes for both staff and residents are more likely to be sustained [58]. An example of this is the LEAP (Learn, Empower, Achieve, Produce) program, which assesses participating facilities' organisational readiness to support their staff in practicing the new approach and skills. However, without an adequately prepared and supported leadership that has the organisational confidence to be assertive [59], the task of change will remain unsustainable and an unsatisfactory experience for many.

The increase in resident morbidity has highlighted the need for clinical leadership for middle managers in residential aged care, yet there are few aged care-specific managerial leadership programs encompassing clinical leadership on offer from educational institutions. In terms of a national approach to aged care leadership, the UK has two well-known programs: an aged care 'leadership and management product' within the Skills for Care program under the umbrella of social care [49], and the Royal College of Nursing (RCN) Clinical Leadership Development program, which was initially funded under the NHS Modernisation Initiative [36]. Whilst both programs emphasise the importance of leadership and management skills, the RCN leadership program targets clinical leaders and practitioners who may or may not be in a management position. This UK RCN based program is also offered in Australia at the Royal Adelaide Hospital, mainly catering to senior clinicians and managers in acute and community care settings with financial support from their organisations [60]. The review suggests that leadership development of RAC managers relies on the motivation of individuals and individual organisations [42,43].

## Development of Policy Options

We took an analytical step to identify potential policy responses using Buse et al.'s approach [61], which considers four key domains in developing and analysing policy: actors (key stakeholders that are influenced by and influencing policy development), context (socio-political and economic factors by which health policy is influenced), content (particular policy elements operating at micro, meso or macro levels), and process (the cycle of developing, communicating, implementing and evaluating policy). Considering those key influential aspects is critical to policy development; otherwise policy ideas will be likely to fall short. The following two sections therefore briefly describe the current climate of the Australian aged care sector necessary for the synthesis and policy development processes.

### Australian aged care policy, regulation and funding

Since 1997 the 'ageing in place' policy has seen resident characteristics in low care facilities become more diverse, necessitating further changes in service and funding arrangements [62]. This policy allows residents to progress from low care to high care in the one facility, depending on the facility's available resources to service higher needs. Since that time, previously low care facilities have needed to develop appropriate accommodation, employ higher skilled staff and conduct culture change programs to adjust staff to both new care models and the more diverse needs of residents.

The clamour for change in care practices and anticipated growth in the aged care sector produced the Aged Care Act 1997 and Quality of Care Standards 1997, with the Resident Classification Scale (RCS) as their funding assessment tool, which was replaced with the Aged Care Funding Instrument (ACFI) in 2008 [63]. Over the next decade, this funding system was felt to be punitive by some and regarded by many as diverting valuable time from care delivery [64]. Aged care has become perhaps the most stringently regulated sector, with the government setting bed numbers by an allocation system on the one hand and the Aged Care Standards and Accreditation Agency Ltd ('the Agency' hereafter) controlling quality standards on the other [6,65]. Currently, RACFs are assessed against the Accreditation Standards. Standard 1 deals with management systems, staffing and organisational development and is assessed with reference to expected outcomes [6,65]. There are guidelines for the Agency's specified areas, which might be extended to include policies and systems in place that ensure cohesive leadership support. However, there is no means by which the effectiveness of individual leadership or an organisation's ability to support its leadership might be formally assessed.

The increased demand on aged care services over the past decade has seen the sector struggle, with a persistent funding shortfall of $513 m per annum between 1997 and 2006 [6]. In 2007 the Federal Government responded to the 2004 Hogan Review of aged care pricing with increases in bed allocations and funding for more training positions and aged care nursing scholarships [66]. However, these increases fell short of Hogan's recommendations as they failed to address "strategic issues bearing upon the implementation of policies to secure efficiencies and quality outcomes in aged care" (p.104) [67]. The 2008 Productivity Commission Report [2] questions whether, given the sector's fragmentation and multiple government departments at multi-tiered levels overlapping in a diverse array of programs, it is possible to genuinely assess needs and services. Yet assessment is essential for the provision of services and perhaps a more reflexive funding system might be developed. Consequently, middle managers responsible for maintaining quality care outcomes with limited funding and adequate skill mix of staff may find themselves ill equipped for appropriate leadership and management skills, the situation being exacerbated by the lack of organisational leadership and adequate policy to support their work as a manager.

### Leadership programs in Australia

Across comparative national health systems, leadership development in aged care has only recently emerged as a critical issue in its own right. Most remain integrated in professional development offerings, and the availability of formal programs focusing on both leadership and management development is patchy.

There are a number of Australian Federal Government initiatives currently available to encourage development of leadership and management in aged care, through awards and additional funding/programs for education and training. Key initiatives include the 'Bringing Nurses back into the Workforce' program; the Aged Care Nursing Scholarship Scheme and the provision of postgraduate scholarships for community aged care nurses; the Support for Aged Care Training Program targeting rural and regional areas; dementia-related education and training; and the Community Aged Care Workforce Development and the 'Better Skills for Better Care' programs. However, the focus of these programs/initiatives has been on rewarding a limited number of managers or facilities for their excellence, or towards training and education for staff involved in direct care and supervision. None of the initiatives provide a systematic and strategic direction for the development of leadership and management roles for middle managers.

In reference to Australian education and training in health care leadership and management, there is no stand-alone nursing leadership and management institute and the national discourse is not as developed as that of other developed countries. Most Australian university nursing schools have some leadership dimensions embedded in the general curriculum, but few leadership-specific courses. Recently several nursing schools in Australia (Flinders University and University of Western Sydney, for example) have started offering postgraduate degrees in aged care specific management. Deakin University School of Nursing offers annual 'Leadership in Nursing' awards to recognise and develop emerging leaders. There are also non-degree management courses offered through vocational training organisations; however they are mostly generic programs such as health management courses.

There is limited information, and certainly no research evidence, on the clinical and managerial leadership development of middle managers driven by the aged care industry in Australia. Anecdotal evidence and consultations with the reference group suggest there is a large gap in terms of aged care industry supported leadership development; an issue that requires a significant investment from the aged care sector.

What has been also found consistently across all sectors, and confirmed by all members of the reference group, is that there is no clear delineation of the roles and responsibilities of types and levels of RAC middle management. The distinction within various types of middle management (e.g., care manager, deputy/director of nursing, care director) can be blurred and their use may even cross between senior and immediate management roles, depending on the way each organisation is structured and operated. This broader use of middle management reflects the current Australian aged care sector, where most middle managers have a nursing background. However, the position is not exclusive to nurses. The review also indicates there is no national database that describes characteristics of the aged care workforce at its management level. Understanding the nature and characteristics of the aged care workforce is important in developing a leadership and management program that is both appropriate to a relevant management level, and in terms of systematic workforce planning and ongoing comprehensive monitoring of the workforce.

### Policy options and strategies

The synthesis processes of the literature, combined with the examination of the Australian aged care context and the consultations, have produced evidence for developing policy options and strategies. Table 3 provides a list of potential policy actions and options necessary for the enhancement of leadership and management for the Australian RAC workforce at an individual manager level, at an organisational level (employer) and at government level.

**Table 3:** Potential policy options and strategies

Key issues	Policy options and strategies arising from the review and consultations
**Education & training**	*Development of an Aged Care Specific Leadership and Management Qualities Framework*, encompassing sets of competencies (addressing the key elements of leadership such as hands-on accessibility and professional expertise in nurturing respect, recognition and team building, along with effective communication and flexibility) and key performance indicators for systematic evaluation. The framework should be congruent with Person Centred Care--the most widely recognised and recommended care philosophy for aged care.
	
	*Development of a leadership and management programme *based on the qualities framework mentioned above. It should be RAC specific, affordable and accessible, allowing for flexibility based on organisational uniqueness and context, and leadership's time constraints.
	
	*Establishment of a partnership approach *in the leadership and management framework and program in collaboration with policy/decision makers, aged care industry representatives, an accreditation body, consumers/carers, education and training organisations.
	
	*Establishment of an aged care leadership and management centre *focused on developing, identifying and disseminating effective educational and training programs and best practices for improving leadership and supervisory skills among people in managerial positions and providing a hub for peer networking and mentoring, and building leadership transmission.

**Regulation, legislation & accreditation**	The relevance of clinical qualifications in aged care needs to be carefully considered, and required only when essential to the role to be performed. Increasingly, middle management in RAC has no requirements for nursing qualifications. It is therefore important for an experienced clinical nurse with the relevant qualifications to be always available in the RACF.

**Incentives, remuneration and reward**	Career paths in administration and senior management to be made available for nurses and other care staff to ensure senior management understand the floor environment.
	
	Development of relevant policies guiding the notion of attractive career paths and succession planning with increased incentives, remuneration and reward; further promotion of career pathways, integration of leader/manager succession planning into organisational culture, and movement between the different leadership/management levels.
	
	Recognition of tertiary leadership and management development qualifications
	
	The image of the RAC industry to be modernised and made more appealing through for example wage parity between acute and non-acute care.

**National minimum dataset (MDS)**	A MDS to be set up for ongoing data collection detailing types of managers, their diversity and the qualifications they hold, pay and remuneration, and turnover and retention. In order to be able to conduct complex and systematic workforce planning and ongoing comprehensive monitoring of the workforce, the establishment of a MDS is necessary.

**Aged care leadership & management strategy**	A national strategy that promotes a common approach to aged care leadership and management development at both government and aged care industry levels. Under this strategy the importance of and access to education and training for leadership and management development can be clearly articulated in relevant policy listed earlier.

This review suggests these policy options are highly relevant internationally, in particular among developed countries such as UK, US, Canada, New Zealand and Scandinavian countries, which share similar problems and concerns associated with the RAC workforce while striving to improve the quality of care they provide for their frail older citizens.

## Discussion and Conclusions

Strengthening of leadership and management skills in the RAC sector is critical in ensuring adequate care quality and the health and well-being of those who receive and provide the care. The essential attributes of good leadership for aged care middle management are a hands-on accessibility and professional expertise in nurturing respect, recognition and team building, along with effective communication and flexibility. However, focusing on individual leadership and management development cannot be a panacea to bring successful and positive outcomes, and any endeavour to improve leadership and management is likely to fail without organisational leadership and appropriate policy that guarantees philosophical cohesion, coherent psychological and structural frameworks, and physical and environmental support (e.g., adequate financial, physical and administrative resources, staffing ratios and skill-mix, remuneration, and staff training and development). The supply of the right workforce for the right job, with clear delineation of scope of practice, appropriate workload and skill mix, and maximum utilisation of the workforce should be ensured so middle managers can be supported in their practice. An important proviso is that any assessment of these capacities would be best framed as supportive and developmental, rather than punitive. The onus is on aged care industries as a whole and various levels of Government to make a concerted effort to establish relevant regulation, legislation and funding.

The Australian RAC sector is undergoing substantial developmental change, much of it for the better, as the Australian Government moves to address some of the most conspicuous workforce issues. The government's workforce initiatives to increase RAC positions and provide more staff training opportunities that include career path opportunities for direct care staff are welcome, as are the increases in aged care scholarships for new and re-entering nurses. However, this review has identified leadership and management as key factors in the delivery of quality care and exposed the absence of attention paid to assuring appropriately trained and qualified leadership and management staff for RACFs. Current leadership and management education and training for nurses and others who are likely to be in a position of middle management is plainly inadequate, yet many of the initiatives now in place to improve staffing and quality for both acute and aged care nursing will depend on good leadership and effective management for their success. What has also been consistently found in the review is that programs are most likely to succeed and be effective when they adopt perennial implementation, run approximately 12 months and are supported through or offered as part of organisational development, rather than programs heavily relying upon individual managers' professional development activities or motivation, without the support of their organisation. If the RAC sector is to meet the impending challenges, urgent attention to the development and promotion of clinical and managerial leadership excellence within middle management is critical.

## Competing interests

The funding organisation had no role in the study design, data collection, analysis and interpretation, or the writing and publication of this article. The authors declare that they have no competing interests.

## Authors' contributions

Y-HJ conceived and designed the study, participated in all stages of literature search, data extraction and synthesis. NJG contributed to the early design concepts, and methods for the review. TM and ES collated and summarised articles. Y-HJ, TM and ES drafted different sections of this paper, and Y-HJ revised the paper as a whole. All authors contributed to the writing and production of the final manuscript.

## Pre-publication history

The pre-publication history for this paper can be accessed here:

http://www.biomedcentral.com/1472-6963/10/190/prepub
